# Molecular Regulatory Networks for Improving Nitrogen Use Efficiency in Rice

**DOI:** 10.3390/ijms22169040

**Published:** 2021-08-21

**Authors:** Mengmeng Hou, Ming Yu, Zhiqiang Li, Zhiyuan Ai, Jingguang Chen

**Affiliations:** 1 School of Agriculture, Sun Yat-sen University, Guangzhou 510275, China; 2 Shenzhen Branch, Guangdong Laboratory of Lingnan Modern Agriculture, Genome Analysis Laboratory of the Ministry of Agriculture and Rural Affairs, Agricultural Genomics Institute at Shenzhen, Chinese Academy of Agricultural Sciences, Shenzhen 518120, China; houmengmeng@caas.cn (M.H.)yuming@caas.cn (M.Y.); 104753200991@vip.henu.edu.cn (Z.L.); 3 State Key Laboratory of Crop Stress Adaptation and Improvement, School of Life Sciences, Henan University, Kaifeng 475004, China; 4 Shenzhen Research Institute of Henan University, Shenzhen 518000, China; 5 State Key Laboratory for Conservation and Utilization of Subtropical Agro-Bioresources, College of Life Science and Technology, Guangxi University, Nanning 530004, China; 2008301001@st.gxu.edu.cn

**Keywords:** nitrogen use efficiency (NUE), absorption and transport, assimilation, nitrate, ammonium, transporter, quantitative trait locus (QTL), hormone, rice (*Oryza sativa* L.)

## Abstract

Nitrogen is an important factor limiting the growth and yield of rice. However, the excessive application of nitrogen will lead to water eutrophication and economic costs. To create rice varieties with high nitrogen use efficiency (NUE) has always been an arduous task in rice breeding. The processes for improving NUE include nitrogen uptake, nitrogen transport from root to shoot, nitrogen assimilation, and nitrogen redistribution, with each step being indispensable to the improvement of NUE. Here, we summarize the effects of absorption, transport, and metabolism of nitrate, ammonium, and amino acids on NUE, as well as the role of hormones in improving rice NUE. Our discussion provide insight for further research in the future.

## 1. Introduction

Rice is the staple food for more than half of the world’s population. Nitrogen is the most abundant mineral element in plants and the main limiting factor in rice production. The application of nitrogen fertilizer has greatly increased global crop yield, accompanied by increasingly severe economic and environmental costs [1]. Therefore, it is urgent to understand the molecular regulation mechanism of nitrogen absorption and utilization in rice and formulate strategies to improve nitrogen use efficiency (NUE). NUE is the guarantee of high yield and quality of rice. With the rapid development of agriculture in China, the input of nitrogen fertilizer has reached significant scale, and some new varieties of rice have demonstrated a problematically low utilization efficiency of fertilizer [1,2]. Today, China is the largest fertilizer consumer in the world, with the production and application of nitrogen fertilizer accounting for more than one third of global total (IFA annual report, 2015). According to statistics, from 1980 to 2013, the net amount of agricultural nitrogen fertilizer application increased by nearly 1.6 times. However, the total grain yield only increased by 87%, the seasonal (traditional) NUE of main crops was only about 35%, and the nitrogen efficiency (including soil residual fertilizer nitrogen) was only about 48%, which was significantly lower than the international average [2,3]. Excessive application of nitrogen fertilizer results in energy waste, higher costs, soil acidification, water eutrophication and greenhouse gas emissions, which seriously restricted the sustainable development of agricultural production [1,4]. Besides, in the pursuit of high yield, how to fully explore the genetic potential of nitrogen absorption and utilization in rice, to reduce the demand for nitrogen from breeding and improve NUE, remains a crucial scientific problem in crop molecular design breeding.

## 2. Genetic Variation in NUE among Various Rice Germplasm

There are significant genetic and environmental differences in nitrogen uptake and utilization in rice. Currently, cultivated rice is mainly divided into two subspecies, Indica and Japonica [5]. Japonica is suitable for high-altitude areas with mild climate, with strong cold and fertilizer tolerance, while Indica is suitable for tropical and subtropical areas with high temperature, strong light and humidity, with weak cold and fertilizer tolerance. Compared with Japonica rice, Indica had higher nitrate uptake and utilization efficiency and overall NUE [6,7,8,9]. In addition, there were significant differences in NUE among local varieties, conventional rice, and hybrid rice, as well as among different varieties or lines within the same subspecies. The study [10] has shown that average nitrogen uptake and utilization efficiency of Indica rice was 14.1% higher than Japonica, and hybrid rice was higher than that of conventional rice. Among them, hybrid Indica rice is 22.8% higher than conventional Indica rice, and hybrid Japonica rice is 16.4% higher than conventional Japonica rice [9]. The genetic diversity of rice nitrogen use efficiency also provides a theoretical basis for the breeding and improvement of NUE in rice varieties.

Based on the significant genetic differences in nitrogen use efficiency among rice germplasm, researchers have used different germplasm resources to construct genetic populations, screening and identifying several QTLs related to nitrogen use efficiency in recent years. *qNUEP-6* was identified to function in NUE through the recombinant inbred line (RIL) population constructed by *Zhenshan 97* and *Minghui 63* [11]. Multiple QTLs related to NUE were subsequently identified by using various RIL and chromosome segment substitution line (CSSL) populations [3,12]. In addition, several QTLs related to low nitrogen tolerance were identified in different genetic populations [13,14]. A total of 110 early rice varieties collected from 52 countries (regions) in different geographical regions of the world were comprehensively identified for agronomic traits. It was found that there was a high correlation between tillering nitrogen response ability and NUE variation in many agronomic traits under different nitrogen fertilizer conditions. A variant of *OsTCP19* promoter was identified by genome-wide association analysis (GWAS). Further study found *OsTCP19* regulated nitrogen uptake by regulating the expression of nitrogen utilization genes to meet the demand of nitrogen growth [2]. A similar situation occurs in the promoter of *OsARE*. After analyzing 2155 rice varieties, we found that 18% of Indica and 48% of Japonica had small insertion in ARE1 promoter, which led to the decrease of *ARE1* expression, caused delayed senescence, and resulted in 10–20% grain yield increases under limited nitrogen condition [15].

## 3. Nitrate Relative Genes Play an Important Role in Improving NUE in Rice

Plants mainly absorb nitrogen from soil in two inorganic forms, nitrate and ammonium. Given the anaerobicity of paddy soil, it is generally believed that ammonium is the main form of nitrogen in rice. However, rice has developed aerenchyma, which can transport oxygen from the ground to the roots and release it into the rhizosphere microenvironment, leaving the rhizosphere soil in a certain oxidation state, thus enhancing the nitrification of soil bacteria to convert ammonium into nitrate, and converting 15–40% of nitrogen into nitrate [16]. A large number of experiments at home and abroad have shown that, compared with single ammonium nitrogen supply, partially nitrate nitrogen can significantly promote the nitrogen absorption and utilization efficiency of rice [17,18,19,20]. Therefore, the uptake and assimilation of nitrate has become an important factor affecting NUE in rice.

In recent years, nitrate transporter genes as a famous family have been used in crop breeding to improve NUE [6,19,21,22,23,24,25,26]. *LeNRT2.3* mediates the low affinity nitrate transport system in tomato. Overexpression of *LeNRT2.3* can increase the uptake of NO_3_^−^ in tomato roots and the transport of NO_3_^−^ from root to shoot, thus increasing the biomass and fruit weight [27]. Tanrt2.1 can increase the uptake of NO_3_^−^ in wheat after flowering [28]. *TaNAC2-5a* is a key transcription factor controlling the expression of nitrate transporter. Overexpression of *TaNAC2-5a* can increase wheat growth, grain yield and nitrogen harvest index [21]. Overexpression of *NAC2* gene in wheat can increase the expression of *TaNRT2.1* and *TaGS2* [21].

**Figure 1:**
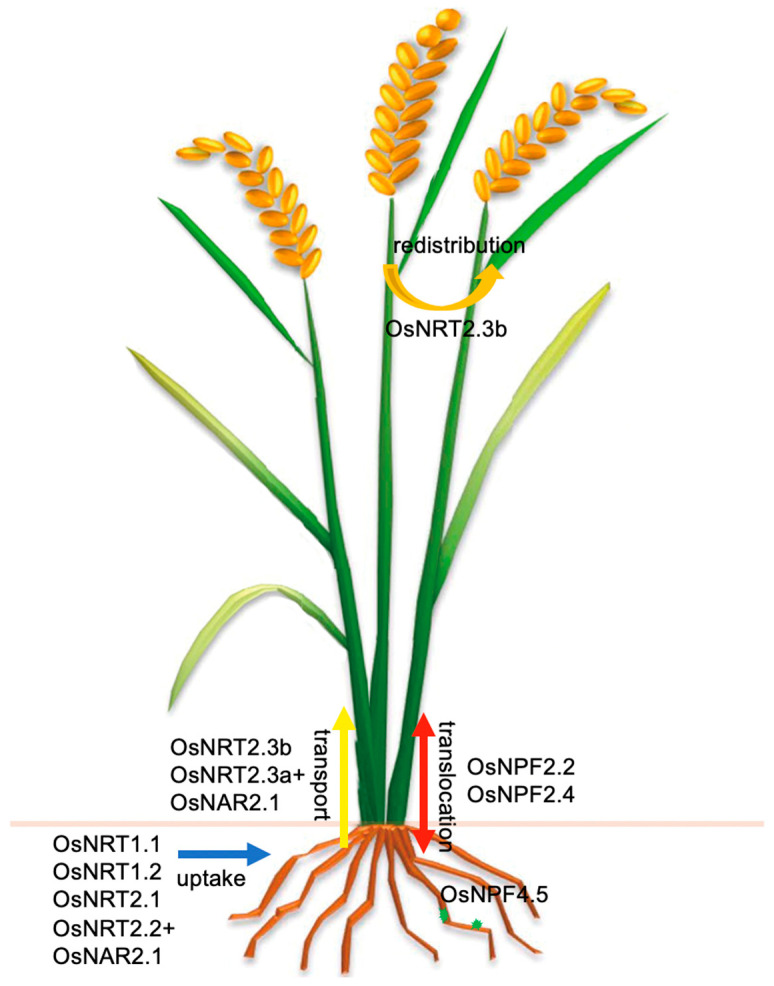
Summary of the contribution of NO_3_^−^ transporters in rice. OsNRT1.1, OsNRT1.2, OsNRT2.1, and OsNRT2.2 (cooperating with OsNAR2.1) are responsible for NO_3_^−^ uptake from the soil. OsNRT2.3a cooperating with OsNAR2.1 is responsible for root-to-shoot NO_3_^−^ transport, and OsNRT2.3b is also responsible for transporting NO_3_^−^ to the shoot and remobilizing N into the grain. OsNPF2.2 and OsNPF2.4 are involved in NO_3_^−^ translocation. OsNPF4.5 is induced by mycorrhizal, involved in symbiotic N uptake. Blue line means NO_3_^−^ uptake, yellow line means transport NO_3_^−^ from root to shoot, red line means NO_3_^−^ translocation, orange line means NO_3_^−^ redistribution.

The absorption and utilization of NO_3_^−^ in rice is a complex process, including absorption, translocation and assimilation. In order to adapt to the changing environment, plants have evolved two nitrate transport systems, low affinity transport systems (LATS) and high affinity transport systems (HATS), which are responsible for NRT1/PTR (NPF) family members and NRT2 family members respectively. The NRT2 family in rice includes *OsNRT2.1*, *OsNRT2.2*, *OsNRT2.3a*, *OsNRT2.3b*, and *OsNRT2.4* [20,23,24,25,29,30,31,32,33]. OsNRT2.1, OsNRT2.2, and OsNRT2.3a need to interact with OsNAR2.1 protein to have NO_3_^−^ transport activity [29,33]. *OsNRT2.3a* was involved in the transport of NO_3_^−^ from root to shoot [34]. A subsequent study found that OsNRT2.3b did not need the interaction of OsNAR2.1 and could transport NO_3_^−^ alone [30]. Overexpression of *OsNRT2.3b* increased the buffering capacity of cell pH and significantly improved the grain yield and NUE of rice [19,26] (Figure 1). Our previous results showed that the expression of *pOsNAR2.1:OsNAR2.1* could increase the NO_3_^−^ uptake rate at seedling stage, significantly promote rice growth, and improve grain yield and agronomic NUE [22]. The expression of *pOsNAR2.1:OsNRT2.1* increased the expression ratio of OsNRT2.1 to OsNAR2.1 in stem, and increased grain yield and NUE in rice [25]. Co-overexpression of *OsNAR2.1* and *OsNRT2.3a* can improve the biomass and nitrate transport efficiency, and ultimately improve the grain yield and NUE in rice [23]. A putative nitrate transporter gene *OsNPF4.5* was induced by mycorrhizal, which is exclusively expressed in the cells containing arbuscules and displayed a low-affinity NO_3_^−^ transport activity involved in symbiotic N uptake [35].

Chlorate is a kind of nitrate analogue, which was in the same pathway as NO_3_^−^ absorption and assimilation in plants. Therefore, chlorate sensitivity simulated nitrate uptake or assimilation in rice. Hu et al. [6] used BC_2_F_5_ lines derived from the cross between IR24 and Nipponbare were screened for chlorate sensitivity, and the chromosome segment substitution line (CSSL) with the highest chlorate sensitivity was used for fine mapping. The nitrate transporter gene *OsNRT1.1b* was cloned from Indica, and then OsNRT1.1b-Indica was introduced into Japonica rice varieties, which could significantly improve the yield and NUE of Japonica rice. *OsNRT1.1b* can also interact with phosphate signal repressor *OsSPX4*, which in turn activates phosphate and nitrate response genes to achieve coordinated utilization of nitrogen and phosphorus [36]. Gao et al. [8] used RIL populations of *9311* and *Nipponbare* to detect a QTL for potassium chlorate resistance, *qCR2*. After fine mapping and cloning, it was found that it encodes a NAD (P) H-dependent nitrate reductase gene *OsNR2*, and the difference between arginine (Indica) and tryptophan (Japonica) in the NAD (P) H binding domain is the determinant of the enzyme activity difference between the two *OsNR2* genes. OsNR2 also interacts with OsNRT1.1b in a positive feedback manner. In Japonica, aggregating these two genes from Indica can obtain higher yield and NUE than a single gene [8]. *OsNPF6.1* was cloned through GWAS analysis based on three-year field experiment, whereby it was found that the excellent haplotype of OsNPF6.1 could enhance nitrogen absorption and NUE, thereby improving rice yield under low nitrogen, and *OsNAC42* can activate the expression of *OsNPF6.1* [37]. OsNPF2.2 is a low-affinity nitrate transporter and participate in unloading nitrate from the xylem [38], OsNPF2.4 functions in acquisition and long-distance transport of NO_3_^−^ [39]. Modified expression of *OsNPF7.1*, *OsNPF7.2*, *OsNPF7.3*, *OsNPF7.4*, and *OsNPF7.7* differentially regulates tillering and grain yield in rice [40,41,42,43]. A NIN-like protein OsNLP4 was identified to function in NUE. Loss of *OsNLP4* dramatically reduced yield and NUE compared with wild type under different N regimes. In contrast, the *OsNLP4* overexpression lines increased yield and NUE under moderate N levels compared with wild type. Transcriptomic analyses revealed that *OsNLP4* orchestrates the expression of a majority of known N uptake, assimilation, and signalling genes by directly binding to the nitrate-responsive cis-element in their promoters to regulate their expression. Moreover, overexpression of *OsNLP4* can rescue the phenotype of *atnlp7* mutant and enhance its biomass [44]. Interestingly, the same results were found through GWAS analysis. *OsNLP4* transactivated *OsNiR* encoding nitrite reductase that was critical in nitrogen assimilation in rice. Using constructed quadrupling NREs (Nitrate-responsive cis-elements) in the promoter of *OsNiR* (*p4xNRE:OsNiR*) enhanced nitrogen assimilation significantly. *OsNLP4-OsNiR* increased tiller number and yield through enhancing nitrogen assimilation and NUE [45]. The nitrate related genes involved in NUE are summarized in Table 1.

## 4. Ammonium, Amino Acid and Nitrogen Assimilation Relative Genes Apply to NUE

Rice grown in paddy fields is an ammonium (NH_4_^+^)-preferring crop. Ammonium is uptaken by ammonium transporters (AMTs) as well as aquaporins or cation channels [61,62,63,64,65,66]. Three members of the rice *OsAMT1* gene family of ammonium transporters showed distinct expression patterns. *OsAMT1;1* was constitutive and ammonium-induced expression in shoots and roots. *OsAMT1;2* was specific expressed in root and ammonium-inducible. *OsAMT1;3* was also root-specific expressed and repressed by nitrogen supply, and glutamine had the same effect on the transcriptional regulation of OsAMT1 genes as ammonium, indicating that glutamine rather than ammonium controls the expression of ammonium transporter genes in rice [62]. *OsAMT1;1* mediates NH_4_^+^ uptake at relatively lower affinity and mediates NH_4_^+^ uptake in a proton independent manner [67]. *OsAMT1;1* significantly contributes to the NH_4_^+^ uptake under both low and high NH_4_^+^ conditions and increased seed yield under suboptimal and optimal N conditions [68,69]. Concomitant activation of *OsAMT1;2* and *OsGOGAT1* genes led to increased tolerance to nitrogen limitation and better ammonium uptake and N remobilization at the whole plant level [70]. Overexpression of *OsAMT1;3* regulates rice growth and carbon-nitrogen metabolic status [71]. Many studies on the genetic manipulation of ammonium uptake of rice in improving NUE are limited. A recent study found that some transcription factors can regulate the expression of ammonium transporters to affect NUE [72]. OsNLP1 protein was localized in the nucleus and its transcription level was rapidly induced by nitrogen starvation. Overexpression of *OsNLP1* increased plant growth, yield and NUE under different nitrogen conditions, while knockout of *OsNLP1* decreased yield and NUE under nitrogen limitation. A further study suggests that *OsNLP1* regulates ammonium and nitrate utilization by coordinating multiple nitrogen uptake and assimilation genes. Chromatin immunoprecipitation (CHIP) and yeast-one hybrid experiments showed that OsNLP1 could directly bind to the promoters of these genes and activate their expression. Overall, enhancing the absorption and remobilization of ammonium will provide a promising strategy to improve rice NUE in the future [73].

It has been proven that plants can obtain organic nitrogen, especially amino acids, through root localized transporters [73,74,75]. When amino acid in soil is relatively high, such as in organic agricultural soil or other planting systems relying on manure or compost for nitrogen nutrition, this absorption system is particularly important [76,77,78]. Some people think that compared with mineral nitrogen fertilizer, the application of high proportion of organic fertilizer can improve the nitrogen absorption, yield, and NUE of crops, and reduce the emission of organic nitrogen to the environment [79]. In rice, the overexpression of *OsAAP1* resulted in increased N uptake and redistribution, as well as significantly increased tiller number and final yield [80]. *OsAAP3* and *OsAAP5* are mainly expressed in the vascular system of rice, and may participate in the transport of amino acids between xylem and phloem [81,82]. RNAi lines of *OsAAP3* and *OsAAP5* also showed significant improvements in tiller number and yield under sufficient nitrogen conditions. Genetic association analyses with 68 representatives Japonica or Indica germplasms identified that *OsLHT1* has a natural variation in aspartate absorption between Japonica and Jndica, and OsLHT1 functions in a broad spectrum of amino acids, and effectively transported aspartate, asparagine, and glutamate in yeast cells. *OsLHT1* is responsible for both the root uptake and root to shoot allocation of a broad spectrum of amino acids in rice [83].

After being absorbed into the plant, the assimilation process is the same except that nitrate needs to be reduced to NH_4_^+^ (NH3). Nitrate is reduced to nitrite in the cytoplasm and transported to the plastid for further reduction to ammonium [84,85,86,87]. The two-step reduction was achieved by nitrate reductase (NR) and nitrite reductase (NIR). NR is the main regulatory step in the process of nitrogen assimilation, and its activity is highly regulated by nitrate, light or water availability [88,89,90,91]. The overexpression of NR or NIR often leads to increased N uptake. Recent studies on rice have shown that the overexpression of Indica *OsNR2* in Japonica can increase tiller number, grain yield, and NUE under high nitrogen supply [8]. This effect was better when Indica *OsNR2* and *OsNRT1.1b* were concurrently expressed, indicating that OsNR2 could regulate *OsNRT1.1b* and thus nitrate uptake [8]. The overexpression of *OsNADH-GOGAT* in rice by its native promoter also resulted in increased grain weight at low N fertilization [92]. Interestingly, the combined overexpression of *OsAMT1;2* and *OsNADH-GOGAT1* in rice can increase NUE in rice under both sufficient and low nitrogen conditions [54]. The results suggest that concurrently increasing N uptake and N assimilation could improve NUE. Some autophagy-related gene could enhance N remobilization and yield and NUE. For example, the overexpression of *OsATG8a* in rice could increase shoot biomass, yield, and NUE when transgenic plants were exposed to high N conditions [93], while overexpression of *OsATG8b* led to increased biomass, yield, and NUE at moderate and low N [94]. Similarly, the constitutive expression of *OsATG8b* in rice led to more biomass and higher grain yield under sufficient N conditions [95], and the overexpression of *OsATG8c* led to improvements in shoot biomass, grain yield, and NUE under moderate and low N fertilization [96].

Too much uptake with a slower assimilation of ammonium may lead to ammonium toxicity and damage to plant growth. Glutamine synthetase is the key enzyme of ammonia assimilation in plants. Ammonium was the main nitrogen source in rice plants, which was assimilated into glutamine and glutamic acid by glutamine synthetase (GS). Glutamine and Glutamic Acid were the fundamental nitrogen sources of other amino acids biosynthesis [97,98,99]. GS has two isomers, cytoplasmic GS1 and plastidic GS2, which have different functions in nitrogen assimilation [100,101]. GS1 isoforms are mainly involved in nitrogen reassimilation and reuse. GS2 participates in primary nitrogen assimilation [102,103]. *OsGS1;1* is involved in ammonium assimilation in rice shoot and root [104]. The overexpression of glutamine synthetase genes *GS1* and *GS2* in transgenic rice plants increases nitrogen-deficiency tolerance [105] and the simultaneous overexpression of *OsGS1* and *OsGS2* genes enhanced the tolerance to osmotic and salinity stress at the seedling stage. There are many examples of using GS gene to improve NUE in multiple species, including Tobacco [106,107,108,109], Arabidopsis [110], Sorghum [111], Maize [112], common wheat [113,114], Barley [115], and Rice [98,116,117]. However, not all GS genes can play a positive role after overexpression, and in some cases, they may have negative effects on plants [106,116,117,118,119,120]. In general, it is possible to use nitrogen assimilation genes to improve the NUE of rice, but this requires the coordination of all aspects of rice development and nutrient condition.

## 5. Plant Hormones Act on NUE

Strigolactones (SLs) have been reported to function in the nutrient-dependent regulation of root and shoot architecture. The biosynthesis mutant *dwarf 10* (*d10*) had a higher N concentration in older leaves but a lower N concentration in younger leaves, while the SL-signaling mutant *dwarf 3* (*d3*) mutant showed a considerably lower N concentration, especially in its younger leaves under normal N levels. Both *d3* and *d10* mutants possess higher N in their leaves under N-deficient conditions. The analysis of uptake and distribution of ^15^N showed that the significant difference of nitrogen concentration between *d3*, *d10,* and WT plants occurred only in leaves, not in roots. The SLS synthesized by exogenous GR24 changed the leaf N distribution of *d10* mutant but did not change the leaf N distribution of *d3* mutant and WT, indicating that the effects of SLS on rice growth and development may be related to the transport of nitrogen to different shoot tissues [121]. AUXIN is a looping star in plant development [122]. A rice nitrogen relative QTL named DULL NITROGEN RESPONSE1 (*qDNR1*) involved in auxin homeostasis, reflects the differences in nitrate (NO_3_^−^) uptake, N assimilation, and yield enhancement between Indica and Japonica rice varieties. Rice plants carrying the DNR1 Indica allele exhibit reduced N-responsive transcription and protein abundance of DNR1 and promotes auxin biosynthesis, inducing AUXIN RESPONSE FACTOR-mediated activation of NO_3_^−^ transporter and N-metabolism genes, resulting in improved NUE and grain yield. Loss-of-function mutation at the DNR1 locus increased N uptake and assimilation, resulting in improved rice yield under moderate levels of N fertilizer input [123]. An auxin efflux carrier *OsPIN9* was induced by ammonium, compared to single nitrate, and involved in ammonium induced rice tillering in rice. The overexpression of *OsPIN9* increased yield under low nitrogen condition [124]. Therefore, appropriate modulation of auxin response is also a promising strategy for achieving NUE in rice. Gibberellin acid (GA) plays a crucial role in agricultural green revolution. The rice *sd1* allele is shorter than normal plants given the reduction of gibberellin, and this increases accumulation of the rice DELLA protein SLR1 (SLENDER RICE1) [125,126,127]. The plants are shorter and therefore more resistant to lodging [128]. Transcription factor *NGR5* (N-mediated tiller growth response 5) is the target of gibberellin receptor *gibberellin insensitive dwarf 1* (GID1), promoting proteasome destruction. NGR5 promotes the N-dependent recruitment of polycomb inhibitor complex 2 through H3K27me3 modification to inhibit branching inhibitors. DELLA protein competitively inhibited GID1–NGR5 interaction, and explained the reason for the tillering increase of green revolution varieties. The increase of NGR5 activity resulted in the decoupling of tillering and nitrogen regulation, thus increasing rice yield at low nitrogen level. Therefore, optimal use of GA could improve the NUE and improve the sustainability of agriculture in the future [129]. Abscisic acid (ABA) is a dominant regulator in plants facing abiotic stress. Osmotic stress/ABA-activated protein kinase 2 (SAPK2) is a member of SnRK2s in rice. The *ossapk2* mutants were shorter and produced fewer grains per panicle, smaller grains, and lower grain yield under drought stress. Moreover, SAPK2 considerably influences the nitrogen contents of rice grains and NO_3_^−^ influx rate, while nitrate concentration analysis indicated that SAPK2 promotes nitrate uptake and assimilation by regulating nitrate-related transporters. These results suggest that ABA related gene could also enhance grain production by regulating NUE under drought stress. Jasmonates (JAs) represent a kind of lipid derived plant hormone, which plays an important role in plant development and defense response to insects. The exogenous addition of methyl jasmonate (MeJA) to rice seedlings significantly reduced root N uptake and ^15^N transport from root to leaf, possibly due to the downregulation of glutamine synthetase and nitrite ferredoxin. MeJA treatment of shoot resulted in the reactivation of endogenous nitrogen from leaf to root. MeJA treatment of root also increased the accumulation of ^14^N in roots, but did not affect the accumulation of ^14^N in leaves. Subsequent analysis of proteomics and RT-qPCR showed that the up regulation of *GDH2*, a plastid disintegration dehydrogenase mediated by JA, contributed to the release of nitrogen in leaves and supported the production of defense proteins/compounds under nitrogen limitation [130]. JA signaling mediates large-scale systemic changes in nitrogen uptake and distribution in rice. Cytokinins affect many aspects of plant growth and development. The overexpression of cytokinin-activation enzyme-like gene *OsLOGL5* significantly reduced primary root growth, tiller number, and yield. On the contrary, a mutation in the C-terminal of OsLOGL5 led to normal plant morphology, but rice yield increased under the conditions of sufficient water, drought, normal nitrogen, and low nitrogen in multiple geographical locations. It was found that the C-terminal of OsLOGL5 protein played an important role in regulating rice yield and improving NUE under different abiotic stresses [131]. In summary, these results suggest that the reasonable use of hormones can also significantly improve the NUE of rice under various growth conditions.

## 6. Future Perspectives

Increasing crop NUE can be achieved by increasing crop yield under the same nitrogen supply or using less nitrogen input for sustainable productivity. A lot of work is now devoted to the identification of candidate genes in improving NUE and their regulatory networks. The basic mechanisms of local and systematic N sensing, uptake, transport, assimilation, and remobilization have been well studied in Arabidopsis, which should be pursued in rice [132]. With the development of bioinformatics, using a high-quality genome sequence, assembly, annotation, and the genome-wide association study (GWAS) of natural genetic variation will further accelerate the discovery of key genes and pathways related to NUE traits in rice. Recently, a new genomic analysis of 33 rice genetic diversity revealed multiple structural variations and gene copy number variations [133]. In order to make use of the variation and phenotype information, researchers should speed up the molecular breeding of these important traits.

**Figure 2:**
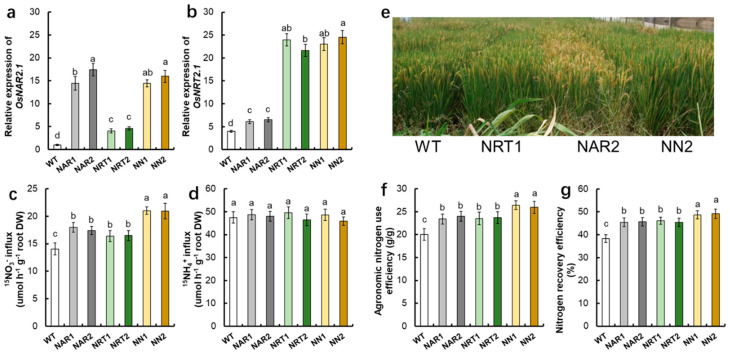
Co-overexpression of *OsNRT2.1* and *OsNAR2.1* increased nitrogen use efficiency in transgenic rice plants. qRT-PCR analysis showed the expression of (**a**) *OsNAR2.1* and (**b**) *OsNRT2.1* of WT, *p35S:OsNAR2.1* transgenic lines (NAR1, NAR2), *p35S:OsNRT2.1* transgenic lines (NRT1, NRT2), and *p35S:OsNAR2.1-p35S:OsNRT2.1* transgenic lines (NN1, NN2). RNA was extracted from root. Error bars: SE (*n* = 3). (**c**,**d**) ^15^N influx rates of transgenic lines. WT and transgenic seedlings were grown in 1 mM NH_4_^+^ for 3 weeks and nitrogen starved for 1 week. ^15^N influx rates were then measured at (**c**) 0.5 mM ^15^NO_3_^−^, (**d**) 0.5 mM ^15^NH_4_^+^ during 5 min. DW, dry weight. (**e**) Photograph of WT and transgenic lines in the field experiment. Comparison of (**f**) agronomic nitrogen use efficiency and (**g**) nitrogen recovery efficiency between the NP and transgenic lines. Error bars: SE (*n* = 4). The different letters indicate a significant difference between the transgenic line and the WT (*p* < 0.05, one-way ANOVA).

Using gene editing by CRISPR-Cas9 technology to change the expression pattern of some genes is a choice for precision agriculture [134]. Nitrate transporters can be effectively used to improve crop yield and through genome editing. Changing the expression of some nitrate transporters or their regulators can improve the NUE in crops [6,19,21,22,23,24,25,135,136]. However, it is not the best way to improve NUE by simply aggregating single positive alleles in rice. The overexpression of multiple genes at the same time can improve the nitrogen use efficiency of more rice. For example, compared with WT, the influx rate of ^15^NO_3_^−^, agronomic nitrogen use efficiency, and nitrogen recovery efficiency of *OsNRT2.1* or *OsNAR2.1* overexpression lines increased by 10%, 20%, and 30%, respectively, while the co-overexpression of *OsNRT2.1* and *OsNAR2.1* lines increased by 40%, 50%, and 60%, respectively (Figure 2). The elite haplotype *OsNPF6.1^HapB^* improves NUE under low nitrogen conditions, which was activated by *OsNAC42* [37]. Indica *OsNR2* promotes NO_3_^−^ uptake by feedback interaction *OsNRT1.1B* (8), which increased effective tiller number, grain yield, and NUE. Therefore, the co-expression of *OsNAC42-OsNPF6.1^HapB^* cascade, Indica *OsNR2-OsNRT1.1B* could improve NUE in rice. In the future molecular breeding, there must be a balance between these genes and pathways, which necessitates better coordination. A potential direction is to find out all possible expression patterns of some useful genes during rice development under various conditions. Using genomic and phenotypic data to explore the best coordinated expression pattern of the regulatory network will deepen our understanding of the determinants for improving NUE. The optimized NUE should meet the best nitrogen demand of rice and be environmentally friendly.

## Figures and Tables

**Table 1:** Candidate NO_3_^−^ transporter and regulation genes for crop application. NUE, nitrogen use efficiency.

Gene	Accession NO.	Species	Characteristic	Nnitrogen Behavior	Reference
*OsTCP19*	Os06g12230	Rice	Responding to nitrate, negatively regulate rice tillering	High tilling response to nitrogen	[2]
*OsNRT2.3b*	AK072215	Rice	Enhancing pH homeostasis, grain yield and NUE	Uptake	[19]
*OsNRT2.3a*	AK109776	Rice	Root-to-shoot NO_3_^−^ transport	Transport	[34]
*OsNAR2.1*	NC_029257.1	Rice	Interaction with OsNRT2.1/2.2/2.3a	Promote uptake and transport after interaction	[29,33]
*OsNRT1.1B*	Os10g40600	Rice	Improving NUE in Indica rice	Uptake and transport	[6]
*OsNPF2.2*	Os12g44100	Rice	Unloading NO_3_^−^ from the xylem and effects on root-to-shoot NO_3_^−^transport	Transport	[38]
*OsNPF2.4*	AK099321.1	Rice	Long-distance NO_3_^−^ transport and regulating NO_3_^−^ and K^+^ shuttle	Uptake and transport	[39]
*OsNRT2.4*	Os01g36720	Rice	Dual-affinity nitrate transporter, nitrate-regulated root growth	Uptake	[32]
*OsNRT1.1A*	Os08g05910	Rice	Regulating N utilization and flowering	Uptake	[20]
*OsNPF4.5*	Os01g54515	Rice	Low-affinity NO_3_^−^ transport activity involved in symbiotic N uptake	Uptake	[35]
*OsNPF6.1*	Os01g01360	Rice	*OsNPF6.1*^HapB^ enhances nitrate uptake and confers high NUE by increasing yield under low nitrogen supply	Uptake	[15]
*OsNPF7.1*	Os07g41250	Rice	regulates tillering and grain yield	Uptake	[40]
*OsNPF7.4*	Os04g50940	Rice	regulates tillering and grain yield	Uptake and transport	[40]
*OsNPF7.2*	Os02g47090	Rice	positively regulates tiller number and grain yield	Uptake and transport	[41]
*OsNPF7.7*	Os10g42870	Rice	regulate shoot branching and nitrogen utilization efficiency	Uptake	[42]
*OsNPF7.3*	Os04g50950	Rice	contributes to nitrogen allocation and grain yield	Translocation	[43]
*OsPTR9*	Os06g49250	Rice	nitrogen utilization efficiency and grain yield	Uptake	[46]
*TaNAC2-5A*	AY625683	Wheat	Positive control of NO_3_^−^ transporter expression and improving NO_3_^−^ accumulation and plant growth	Regualating TaNRT2.5 Enhance nitrate uptake	[21]
*TaNRT2.1*	AF332214	Wheat	NO_3_^−^ uptake at post-flowering stage	Uptake	[28]
*MtNRT2.1*	Medtr4g104730	M. truncatula	Nitrate transport with HATS activity	Uptake	[47]
*MtNRT2.3*	Medtr4g057865	M. truncatula	Controlling post-inoculation processes in nodule functioning	Transport	[47]
*MtNIP/LATD*	GQ401665	M. truncatula	Controlling nodulation and root architecture	Uptake	[48]
*LeNRT2.3*	AY038800	Tomato	NO_3_^−^ uptake and long-distance transport	Uptake and transport	[27]
*CmNRT2.1*	KT203959.1	Chrysanthemum	Enhancing N uptake	Uptake	[49]
*AtNRT1.1*	AT1G12110	Arabidopsis	Directing root growth in sensing external NO_3_^−^ concentration	Uptake	[50]
*AtNPF7.3/AtNRT1.5*	AT1G32450	Arabidopsis	Low NO_3_^−^ dependent K^+^ translocation from root to shoot	Transport	[51,52]
*AtNRT1.11*	AT1G52190	Arabidopsis	Phloem-specific NO_3_^−^ transporter redistributing xylem-borne NO_3_^−^ to enhance plant growth	Transport	[53]
*AtNRT1.12*	AT3G16180	Arabidopsis	Phloem-specific NO_3_^−^ transporter redistributing xylem-borne NO_3_^−^ to enhance plant growth	Transport	[53]
*AtNPF2.3*	AT3G45680	Arabidopsis	NO_3_^−^ excretion transporter and contribution to NO_3_^−^ translocation to the shoot	Transport	[54]
*AtNPF3.1*	AT1G68570	Arabidopsis	Encoding pathogen-inducible NO_3_^−^/NO_2_^−^ transporters	Uptake	[55]
*AtNPF5.5*	AT2G38100	Arabidopsis	Affecting N accumulation in Arabidopsis embryo	Uptake	[56]
*AtNRT2.5*	AT1G12940	Arabidopsis	Loading NO_3_^−^ into phloem of N-starved adult plants	Uptake andtransport	[57]
*AtNRT2.7*	AT5G14570	Arabidopsis	Seed-specific NO_3_^−^ transporter for accumulation/oxidation of proanthocyanidins	Transport	[58]
*AtNLP7*	AT4G24020	Arabidopsis	Enhancing plant growth in N-sufficient conditions	Sensing and assimilation	[59]
*AtHY5*	AT3G17609	Arabidopsis	Binding NRT2.1 promoter	Activating NRT2.1 expression and nitrate uptake	[60]

## Data Availability

All data generated or analyzed during this study are included in this published article.
